# High frequency of the *PIK3CA* H1047L mutation in Indonesian breast cancer across molecular subtypes

**DOI:** 10.1371/journal.pone.0322154

**Published:** 2025-05-05

**Authors:** Yan Wisnu Prajoko, Didik Setyo Heriyanto, Bayu Tirta Dirja, Susanto Susanto, Vincent Lau, Andrew Nobiantoro Gunawan, Brigitta Natasya Halim, Nur Dina Amalina

**Affiliations:** 1 Department of Surgical Oncology, Faculty of Medicine, Universitas Diponegoro, Semarang, Indonesia; 2 Department of Anatomical Pathology, Faculty of Medicine, Public Health and Nursing, Universitas Gadjah Mada/Dr. Sardjito General Hospital, Yogyakarta, Indonesia; 3 Department of Surgery, Division of Cardiac, Thoracic, and Vascular Surgery, Faculty of Medicine, Public Health, and Nursing/Dr. Sardjito General Hospital, Yogyakarta, Indonesia; 4 Collaboration Research Center for Precision Oncology based Omics- PKR PrOmics, Yogyakarta, Indonesia; 5 Department of Microbiology, Faculty of Medicine, Mataram University, Mataram, Indonesia; 6 Semarang Medical Center (SMC) Telogorejo Hospital, Semarang, Indonesia; 7 Department of Pharmaceutical Sciences, Faculty of Medicine, Universitas Negeri Semarang, Semarang, Indonesia; University of Alabama at Birmingham, UNITED STATES OF AMERICA

## Abstract

Breast cancer (BC) is a global health concern with significant mortality rates, necessitating a deep understanding of its molecular landscape. Luminal A and B BC, characterized by estrogen receptor (ER) and/or progesterone receptor (PR) positivity, face challenges in endocrine therapy due to acquired resistance, frequently driven by PI3K/AKT/mTOR pathway activation. This study focuses on the frequency of *PIK3CA* mutations across molecular subtypes BC within the Indonesian population. The study analyzed collected samples from diverse Indonesian regions, representing various islands. Histopathological analysis and immunohistochemistry classified samples into molecular subtypes. Genetic analysis using *PIK3CA* mutation detection kits revealed a mutation frequency of 32.9%, with 30 (14.5%) samples located in exon 9 and 38 (18.4%) samples in exon 20. Statistical analyses highlighted associations between *PIK3CA* mutations and molecular subtypes (p = 0.029), with luminal B HER2-negative (40.5%) and luminal A (40.2%) exhibiting the highest mutation rate. A significant association was also observed between the exon location of only mutated *PIK3CA* samples and age group (p < 0.001), with most of the *PIK3CA* exon 9 being ≤ 50 years old (72.4%) and *PIK3CA e*xon 20 being > 50 years old. No statistically significant association was observed between the location of *PIK3CA* mutation (exons 9 and 20) and the breast site, histopathological diagnosis, and molecular subtypes. Comparisons with existing literature and inconsistencies in *PIK3CA* mutation frequencies across different BC subtypes underline the need for population-specific research. The study emphasizes the importance of assessing *PIK3CA* mutations in BC management, offering insights for personalized therapies and potential advancements in understanding this complex disease within the Indonesian context.

## Introduction

Breast cancer (BC) is a significant global public health issue with a high mortality rate among women [1,2]. It is the second most diagnosed cancer after lung cancer, with over two million new cases and the fourth leading cause of cancer-related deaths for both sexes in 2022 [3]. Statistically, 5–10% of BCs are caused primarily by genetic factors caused by the accumulation of acquired somatic changes [4,5] The diagnosis, prognosis, predictive usefulness, therapy, and prevention of BC depend on molecular biomarkers and their association with pathological features [6].

Research has underscored the significance of analyzing phosphatidylinositol-4,5-bisphosphate 3-kinase catalytic subunit alpha (*PIK3CA*) gene mutations due to their association with the onset and progression of BCs. These mutations are observed in 20–40% of BC patients [7–10]. Positioned on chromosome 3q26.32, the *PIK3CA* gene encodes the alpha isoform (p110α), the principal isoform of the catalytic subunit within the class 1A PI3K, a lipid phosphokinase [7,9,11]. Functionally, the PI3K family mediates signals crucial for various cellular activities, encompassing proliferation, metabolism, migration, translation, apoptosis evasion, and angiogenesis. Four common ‘hotspot’ *PIK3CA* mutations (E542K, E545K, H1047R, and H1047L) constitute 80–90% of all *PIK3CA* mutations in human cancers and serve as predictive biomarkers [7,11,12].

Previous research has indicated that endocrine-resistant *PIK3CA*-mutant cases may potentially benefit from treatment with PI3K inhibitors, underscoring the significance of elucidating the prevalence of *PIK3CA* mutations among populations to inform potential future avenues in hormone receptor-positive BC treatment [13–15]. Research also suggests that the presence of *PIK3CA* mutations can adversely affect the disease-free survival (DFS) and pathological complete response (pCR) to targeted therapy and chemotherapy in patients with human epidermal growth factor receptor 2 (HER2)-enriched and triple negative breast cancer (TNBC) [7–9,11,16]. This suggests that it might be beneficial to assess *PIK3CA* mutations in all BCs molecular subtypes including the non-hormonal subtypes. However, there is a noticeable lack of data on this subject, particularly in the Indonesian population. So far, many studies have been conducted on BC *PIK3CA* mutations in various countries and regions, and the results of studies related to the clinical pathological characteristics of *PIK3CA* mutation have inconsistencies between one study and another [17–24]. Small sample sizes, different detection methods, and different inclusion criteria are likely to be contributing factors to this inconsistent outcome.

This study aims to determine the frequency of *PIK3CA* mutations among Indonesian BC patients–an underrepresented group in current research–and to examine the association of those mutations with clinicopathological features. Given the limited national data on *PIK3CA* mutations, our findings may contribute to improved BC management in Indonesia and provide a foundation for future research and targeted therapeutic strategies

## Methods

### Study design

This study employed a cross-sectional approach to analyze samples obtained from the archive of Cito Clinical Laboratory, Indonesia, spanning the period from 2019 to 2022. The samples were collected from various islands across the Indonesian archipelago, including Java, Kalimantan, Sumatera, Mataram, and Papua. Histopathological examination and grading of BC samples were conducted, as necessary. Subsequently, the samples underwent immunohistochemistry (IHC) analysis to determine their molecular subtypes. Finally, genetic analysis was performed on each viable sample to identify the presence of any *PIK3CA* mutations. The archival samples for this study were accessed and analyzed from 06/02/2023–06/11/2023.

### Ethics approval

Ethical approval for this study was granted by the Medical and Health Research Ethics Committee (MHREC) under Ethical Approval Number 32/EC/KEPK/FK-UNDIP/II/2023.

### Samples collection

Paraffin blocks obtained from patients histopathologically diagnosed with primary BC, including Invasive of No Special Type (NST), Invasive Lobular Carcinoma, Invasive Papillary Carcinoma, and Mucinous Carcinoma, were included in this study. Samples were collected from multiple centers situated in various provinces across the diverse islands of Indonesia. Initial diagnoses and tumor grading for each sample were obtained from the referring hospitals across Indonesia. Upon receipt at Cito Clinical Laboratory, Yogyakarta, two pathologists, at the institution verified and confirmed the histopathological diagnoses and grades, also assess the quality of paraffin blocks before proceeding with any additional examinations. Exclusion criteria comprised formalin-fixed paraffin embedded (FFPE) samples of inadequate quality and incomplete medical records data. Patient data regarding clinicopathological characteristics, including sociodemographic (age), tumor pathology (location/site, histopathology, histologic grade), were acquired from medical records obtained from referring hospitals.

### IHC staining

Confirmed samples then underwent IHC analysis at Cito Clinical Laboratory and the Department of Anatomical Pathology, Faculty of Medicine, Public Health, and Nursing, Universitas Gadjah Mada, located in Yogyakarta. IHC was utilized to examine the samples, by using anti-estrogen receptor (anti-ER), anti-progesterone receptor (anti-PR), anti-human epidermal growth factor receptor 2 (anti-HER2), and anti-Ki67 antibodies from Ventana™ (Roche Diagnostics, AZ, USA). Staining procedures were conducted utilizing the Ventana BenchMark XT and Discovery XT™ Tissue Diagnostic and IHC Autostainer (Roche Diagnostics, AZ, USA). All protocols strictly adhered to the manufacturer’s instructions and protocols to ensure the attainment of consistent and reliable results. Our institution adheres to the 2013 St. Gallen International Expert Consensus guidelines. According to these guidelines, luminal A BC is defined by the presence of ER and PR, with PR expression at 20% or higher, absence of HER2 expression, and low Ki-67 expression levels (less than 14%). In contrast, luminal B (HER2-negative) BC is characterized by the presence of ER, absence of HER2 expression, PR expression below 20%, or high Ki-67 expression levels. Overexpression or amplification of HER2 (3+; strong reactivity in ≥ 10% of tumor cells in the sample) are defined as HER2-enriched. Meanwhile, lacking ER, PR, and HER2 expressions are defined as TNBC subtypes.

### DNA extraction

Paraffin blocks from various BC subtypes were cut into 10 pieces with 5 μm thickness. Ten pieces in one slide continued with DNA extraction. Genomic DNA was extracted using the GeneAll® Exgene™ FFPE Tissue DNA (GeneAll Clinic SV mini, Seoul, Korea) according to the manufacturer’s protocol. DNA samples were then quantified using a NanoDrop™ spectrophotometer (Thermo Fisher Scientific, Waltham, MA, USA). Samples of sufficient concentration and quality were adjusted to a concentration of 20 ng/ µL for Polymerase Chain Reaction (PCR) applications.

### Detection of *PIK3CA* mutation

The samples were further analyzed for *PIK3CA* mutation at both Cito Clinical Laboratory and the Department of Anatomical Pathology, Faculty of Medicine, Public Health, and Nursing, Universitas Gadjah Mada. Specimens originating from islands other than Java were meticulously transported to Yogyakarta, ensuring strict adherence to established standards. The detection of *PIK3CA* mutation biomarkers was conducted using the BioColomelt-Dx™ in vitro diagnostics kit, developed by Biofarma Ltd, Indonesia. This molecular diagnostic kit utilizes PCR and High-Resolution Melting (HRM) techniques. Subsequently, the findings were validated using the Qiagen Therascreen® *PIK3CA* RGQ PCR kit (QIAGEN, Hilden, Germany). The Qiagen Therascreen® *PIK3CA* RGQ PCR kit (QIAGEN, Hilden, Germany) was designed to detect 11 mutations in exons 7, 9, and 20 of the *PIK3CA* gene. The specific mutations detectable by this kit include C420R in exon 7; E542K, E545A, E545D (1635G > T only), E545G, E545K, Q546E and Q546R in exon 9; and H1047L, H1047R, and H1047Y in exon 20.

### Statistical analysis

The statistical analysis was conducted using Microsoft Excel (Microsoft 365, Washington, USA) and IBM SPSS version 27.0 (IBM, New York, USA). The association between variables was calculated using Fisher’s Exact Test with a significance level of p-values < 0.05.

## Results

### Clinicopathologic characteristics of the BC samples

This study included 207 cases of diverse BC types, with 63.8% of patients aged over age 50 (Table 1). Tumor location, histopathological types, and molecular subtypes are detailed in Table 1, along with their respective frequencies and percentages.

**Table 1:** Clinicopathology characteristics of the study samples.

Characteristic	N (%)
Age Group	
≤ 50 years old	75 (36.2%)
> 50 years old	132 (63.8%)
Location	
Right Breast	104 (50.2%)
Left Breast	103 (49.8%)
Histopathology Diagnosis	
Invasive Breast Carcinoma of NST, Grade I	5 (2.4%)
Invasive Breast Carcinoma of NST, Grade II	84 (40.6%)
Invasive Breast Carcinoma of NST, Grade III	80 (38.6%)
Invasive Lobular Carcinoma of the Breast	33 (15.9%)
Invasive Papillary Carcinoma of the Breast	2 (1%)
Mucinous Carcinoma of the Breast	3 (1.4%)
Molecular Subtypes (IHC Evaluation)	
Luminal A	102 (49.3%)
Luminal B HER2-negative	37 (17.9%)
Luminal B HER2-positive	21 (10.1%)
HER2-enriched	20 (9.7%)
TNBC	27 (13%)
Total	207 (100%)

NST = No Special Type, IHC = Immunohistochemistry, HER2 = Human Epidermal Growth Factor 2, TNBC = Triple Negative Breast Cancer.

### *PIK3CA* mutations landscape in BC samples

**Fig 1:**
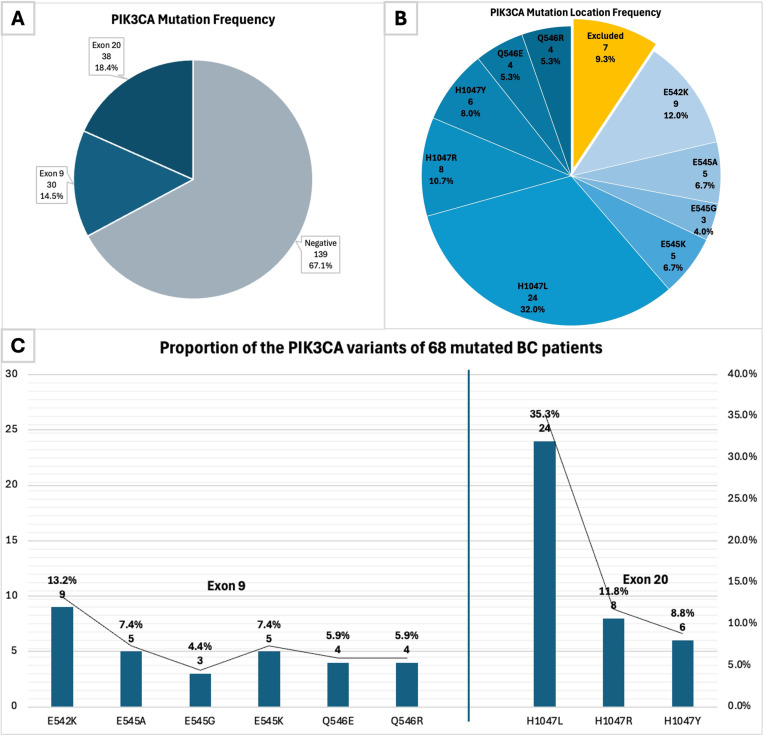
*PIK3CA* mutation. (A) *PIK3CA* mutation frequency based on the exon location. (B) *PIK3CA* specific mutation spectrum. (C) Proportion of *PIK3CA* variants of all positive *PIK3CA* mutations divided according to exon location.

A total of 68 (32.9%) of 207 samples showed positive *PIK3CA* mutation; 30 (14.5%) samples are located in exon 9 and 38 (18.4%) samples are in exon 20 (Fig 1A). The specific mutation spectrum among BC samples is presented in Fig 1B. The 7 (9.3%) samples that are identified for positive *PIK3CA* mutation using BioColomelt-Dx™ in vitro diagnostics kit (Biofarma Ltd, Jakarta, Indonesia) yet negative in Qiagen Therascreen® *PIK3CA* RGQ PCR kit (QIAGEN, Hilden, Germany) (Fig 1B) are subsequently excluded from the association analysis. The most common variants in our samples are H1047L (24, 35.3%) for exon 20 and E542K (9, 13.2%) for exon 9 (Fig 1C).

The association of *PIK3CA* mutation status and exon location with clinicopathological characteristics is summarized in Table 2. Overall, no significant differences were noted in *PIK3CA* mutation frequency by age group (≤ 50 vs. > 50 years) or tumor location (OR 0.665, 95% CI 0.366–1.208, p = 0.218; and OR 1.209, 95% CI 0.676–2.160, p = 0.556, respectively). However, among tumors already harboring a *PIK3CA* mutation, patients aged ≤ 50 years were markedly more likely to have an exon 9 mutation than exon 20 (OR 8.750, 95% CI 2.902–26.382, p < 0.001). In terms of molecular subtype, *PIK3CA* mutations were significantly associated with luminal B HER2-negative tumors (p = 0.029), which demonstrated the highest mutation rate (40.5%), followed by luminal A (40.2%), HER2-enriched (20%), TNBC (18.5%), and luminal B HER2-positive (14.3%).

**Table 2:** Association between *PIK3CA* mutation and mutation exon locations with clinicopathological characteristics of BC.

Characteristics	Number of patients	*PIK3CA* mutation		OR (95% CI)^a^	*P*-value	*PIK3CA* mutation exon locations		OR (95% CI)^a^	*P*-value
		Negative N (%)	Positive N (%)			Exon 9 N (%)	Exon 20 N (%)		
Age Group				0.665 (0.366 – 1.208)	0.218			**8.750 (2.902** – **26.382)**	**<0.001** ^*^
≤ 50 years old	75	46 (61.3%)	29 (38.7%)			21 (72.4%)	8 (27.6%)		
> 50 years old	132	93 (70.5%)	39 (29.5%)			9 (23.1%)	30 (76.9%)		
Location				1.209 (0.676 – 2.160)	0.556			0.765 (0.292 – 2.000)	0.631
Right Breast	104	72 (69.2%)	32 (30.8%)			13 (40.6%)	19 (59.4%)		
Left Breast	103	67 (65%)	36 (35%)			17 (47.2%)	19 (52.8%)		
Histopathology Diagnosis					0.255				0.709
Invasive Breast Carcinoma of NST, Grade I	5	5 (100%)	0 (0%)			0 (0%)	0 (0%)		
Invasive Breast Carcinoma of NST, Grade II	84	56 (66.7%)	28 (33.3%)			14 (50%)	14 (50%)		
Invasive Breast Carcinoma of NST, Grade III	80	49 (61.3%)	31 (38.7%)			13 (41.9%)	18 (58.1%)		
Invasive Lobular Carcinoma of the Breast	33	25 (75.8%)	8 (24.2%)			3 (37.5%)	5 (62.5%)		
Invasive Papillary Carcinoma of the Breast	2	1 (50%)	1 (50%)			0 (0%)	1 (100%)		
Mucinous Carcinoma of the Breast	3	3 (100%)	0 (0%)			0 (0%)	0 (0%)		
Molecular Subtypes (IHC Evaluation)					**0.029** ^*^				0.929
Luminal A	102	61 (59.8%)	41 (40.2%)			17 (41.5%)	24 (58.5%)		
Luminal B HER2-negative	37	22 (59.5%)	15 (40.5%)			7 (46.7%)	8 (53.3%)		
Luminal B HER2-positive	21	18 (85.7%)	3 (14.3%)			2 (66.7%)	1 (33.3%)		
HER2-enriched	20	16 (80%)	4 (20.0%)			2 (50%)	2 (50%)		
TNBC	27	22 (81.5%)	5 (18.5%)			2 (40%)	3 (60%)		

*Fisher’s Exact Test of p < 0.05. CI = Confidence Interval, HER2 = Human Epidermal Growth Factor 2, IHC = Immunohistochemistry, NST = No Special Type, OR = Odds Ratio, TNBC = Triple Negative Breast Cancer.

^a^Odds ratio and 95% confidence intervals were calculated using the risk estimate method from a 2x2 contingency table.

Further exploration on the specific *PIK3CA* mutation location with clinicopathological characteristics are presented in Table 3. A significant association between *PIK3CA* specific mutation location and age group is observed (p < 0.001), consistent with previous *PIK3CA* mutation exon location (p < 0.001, Table 2). Patients >50 years old have higher frequency of H1047L and H1047R mutations.

**Table 3:** Association between specific *PIK3CA* mutation location and clinicopathological characteristics.

Characteristics	Number of Patients	Specific *PIK3CA* Mutation Location									*P*-value
		Exon 9						Exon 20			
		E542K	E545A	E545G	E545K	Q546E	Q546R	H1047L	H1047R	H1047Y	
Age Group											<0.001*
≤ 50 years old	29	8 (27.6%)	5 (17.2%)	2 (6.9%)	4 (13.8%)	0 (0%)	2 (6.9%)	6 (20.7%)	1 (3.4%)	1 (3.4%)	
> 50 years old	39	1 (2.6%)	0 (0%)	1 (2.6%)	1 (2.6%)	4 (10.3%)	2 (5.1%)	18 (46.2%)	7 (17.9%)	5 (12.8%)	
Location											0.368
Right Breast	32	6 (18.8%)	1 (3.1%)	2 (6.3%)	2 (6.3%)	0 (0%)	2 (6.3%)	14 (43.8%)	3 (9.4%)	2 (6.3%)	
Left Breast	36	3 (8.3%)	4 (11.1%)	1 (2.8%)	3 (8.3%)	4 (11.1%)	2 (5.6%)	10 (27.8%)	5 (13.9%)	4 (11.1%)	
Histopathology Diagnosis											0.528
Invasive Breast Carcinoma of NST, Grade I	0	0 (0%)	0 (0%)	0 (0%)	0 (0%)	0 (0%)	0 (0%)	0 (0%)	0 (0%)	0 (0%)	
Invasive Breast Carcinoma of NST, Grade II	28	5 (17.9%)	3 (10.7%)	0 (0%)	2 (7.1%)	1 (3.6%)	3 (10.7%)	8 (28.6%)	4 (14.3%)	2 (7.1%)	
Invasive Breast Carcinoma of NST, Grade III	31	3 (9.7%)	0 (0%)	3 (9.7%)	12 (38.7%)	3 (9.7%)	1 (3.2%)	12 (38.7%)	4 (12.9%)	2 (6.5%)	
Invasive Lobular Carcinoma of the Breast	8	1 (12.5%)	2 (25%)	0 (0%)	0 (0%)	0 (0%)	0 (0%)	3 (37.5%)	0 (0%)	2 (25%)	
Invasive Papillary Carcinoma of the Breast	1	0 (0%)	0 (0%)	0 (0%)	0 (0%)	0 (0%)	0 (0%)	1 (100%)	0 (0%)	0 (0%)	
Mucinous Carcinoma of the Breast	0	0 (0%)	0 (0%)	0 (0%)	0 (0%)	0 (0%)	0 (0%)	0 (0%)	0 (0%)	0 (0%)	
Molecular Subtypes (IHC Evaluation)											0.276
Luminal A	41	2 (4.9%)	2 (4.9%)	3 (7.3%)	4 (9.8%)	4 (9.8%)	2 (4.9%)	16 (39%)	5 (12.2%)	3 (7.3%)	
Luminal B HER2-negative	15	1 (6.7%)	3 (20%)	0 (0%)	1 (6.7%)	0 (0%)	2 (13.3%)	5 (33.3%)	2 (13.3%)	1 (6.7%)	
Luminal B HER2-positive	3	2 (66.7%)	0 (0%)	0 (0%)	0 (0%)	0 (0%)	0 (0%)	1 (33.3%)	0 (0%)	0 (0%)	
HER2-enriched	4	2 (50%)	0 (0%)	0 (0%)	0 (0%)	0 (0%)	0 (0%)	2 (50%)	0 (0%)	0 (0%)	
TNBC	5	2 (40%)	0 (0%)	0 (0%)	0 (0%)	0 (0%)	0 (0%)	0 (0%)	1 (20%)	2 (40%)	

*Fisher’s Exact Test of p < 0.05. HER2 = Human Epidermal Growth Factor 2, IHC = Immunohistochemistry, NST = No Special Type, TNBC = Triple Negative Breast Cancer.

## Discussion

The PI3K pathway plays a pivotal role in the proliferation and viability of malignant cells and is frequently dysregulated in various types of cancer, including BC. This deregulation can occur through several mechanisms, such as mutations or amplifications in PI3K itself, the inactivation of the tumor suppressor *PTEN*, or the activation of upstream oncogenes and tyrosine kinase growth factor receptors [25]. *PIK3CA* mutations have been extensively studied in newly diagnosed BC, often linked to favorable characteristics like positive ER expression, smaller tumor size, and low histological grade [26,27]. Studies also found that *PIK3CA* mutations were associated with older age and lower tumor grade at diagnosis [26,28,29]. However, these mutations show variable frequencies and implications across different studies and populations. Recent studies from different populations have also presented conflicting results regarding the relationship between *PIK3CA* mutations and tumor grading, adding the layer of complexity [30]. *PIK3CA* wild-type tumors had lower tumor grade, higher ER expression, and lower androgen receptor (AR) expression compared to mutant tumors, contradictory with recent findings that there was no significant correlation between histological Scarff-Bloom-Richardson (SBR) tumor grading and *PIK3CA* mutation status [31,32]. In contrast, our study is in accordance with the study by Cizkova that showed that *PIK3CA* mutations are predominantly found in higher-grade tumors [7]. Due to the inconclusive findings, additional investigation is warranted, ideally encompassing a larger sample size and more comprehensive population representation.

Our findings indicate a varying frequency of *PIK3CA* mutation across different molecular subtypes, with the highest proportion frequency observed in luminal B HER2-negative, followed by luminal A, HER2-enriched, TNBC, and luminal B HER2-positive subtypes. The results are partially consistent with the research conducted by Wu et al. (2019) [17]. Nonetheless, the study did not further categorize luminal B into luminal B HER2-negative or luminal B HER2-positive [17]. Within the luminal subtypes, mutation rates can reach up to 46.6% in luminal A [33] and approximately 27% in luminal B [34]. In HER2-positive disease, especially the HER2-enriched subtype—which is linked to aggressive behavior and poorer outcomes—about 21% of tumors harbor *PIK3CA* mutations [35]. By contrast, TNBC generally exhibits a lower frequency, varying from 12.5% to as high as 28.6% [22,36]. These distributions reinforce the stronger association of *PIK3CA* mutations with hormone receptor–positive tumors—often tied to more favorable outcomes [37,38]—whereas TNBC cases may experience poorer prognosis [39].

Recent studies have highlighted the frequency of *PIK3CA* mutations in various cancer types within the Indonesian population. For instance, a study by Heriyanto reported a mutation frequency of 43.85% in colorectal cancer patients, which is notably lower than other regions in Indonesia that reported frequencies as high as 70.9% [40]. This discrepancy may be attributed to differences in sample sizes and population genetics, suggesting that the H1047L mutation could be more prevalent in certain demographic groups or cancer subtypes. The implications of these findings are profound, as they suggest that the high frequency of *PIK3CA* mutations, particularly H1047L, could serve as a potential biomarker for targeted therapies in the Indonesian population. Understanding the mutational landscape of *PIK3CA* in this context not only aids in the development of personalized treatment approaches but also enhances our comprehension of the molecular mechanisms underlying cancer in diverse populations [41,42].

Research has shown that *PIK3CA* mutations, including H1047L, can influence treatment responses. For instance, patients with ER + BC harboring *PIK3CA* mutations have shown favorable response to PI3K inhibitors such as alpelisib [15,43]. The prognostic implications of *PIK3CA* mutations, particularly H1047L, appear to be multifaceted. While some studies suggest that these mutations correlate with poorer responses to chemotherapy and targeted therapies, others indicate that they may be associated with favorable outcomes in specific contexts, such as in early-stage, hormone receptor-positive BC [44]. For example, a meta-analysis indicated that *PIK3CA* mutations could serve as favorable prognostic biomarkers in operable BC, suggesting that the clinical significance of these mutations may vary depending on the tumor’s molecular subtype and the treatment regimen [45].

On the other hand, a study highlighted that *PIK3CA* mutations were identified in 24.2% of HER2-enriched breast tumors, with a significant association with poor prognosis in certain contexts [46]. Moreover, the H1047L mutation, along with other hotspot mutations like H1047R, has been linked to the activation of the PI3K signaling pathway, which plays a crucial role in cell proliferation and survival [47,48]. This activation can lead to resistance against various therapies, including trastuzumab and other anti-HER2 agents, thereby complicating treatment outcomes [36,39,49]. Since our results showed a 35.3% frequency of the H1047L mutation, the therapeutic strategy should be discussed by a multidisciplinary team to consider potential drug resistance and guideline implementation.

Therapeutically, the presence of *PIK3CA* mutations has been linked to reduced rates of achieving pCR in neoadjuvant chemotherapy, indicating potential chemoresistance [39,50]. Moreover, these mutations have been associated with resistance to anti-HER2 treatment in HER2-enriched patients and endocrine treatment in ER-positive patients [14,36,51] A previous study reported that ER-positive, HER2-negative, *PIK3CA* mutant BCs, despite apparent PI3K/AKT pathway activation, downstream mTOR1 signaling was not greatly elevated at the transcriptional and biological levels. One of their hypotheses for underlying the mechanism is that *PIK3CA* mutations are associated with weak pathway activation, and that other PI3K pathway alterations produce stronger pathway activation. The study suggests that, in ER-positive/HER2-negative BC with *PIK3CA* mutations, pathway activation surprisingly does not result in greatly elevated downstream signaling and their functional output differs substantially compared with that of *PTEN* loss [50,52].

Notably, the C420R mutation is not identified in any of our samples. The mutation, while less common than the major hotspots such as H1047R and E545K, has been shown to possess oncogenic properties comparable to these more prevalent mutations [53]. This suggests that even less frequent mutations like C420R, representing 1–1.9% of all *PIK3CA* mutations, can impact tumor biology and patient outcomes [10,54].

Approximately 30–40% of BC cases that are HER2-enriched exhibit a mutation in the *PIK3CA* gene [55,56]. Some studies have shown that the *PIK3CA* mutation in circulating tumor DNA (ctDNA) was observed in patients who tested negative for the mutation in the tissue samples [57,58]. As a result, the implementation of liquid biopsy as a valuable method to more effectively capture temporal heterogeneity and detect metastatic disease has been proposed in various other studies [58–61]. This approach has the potential to increase the number of patients who may derive therapeutic benefits from targeted treatments, since such mutations have been associated with trastuzumab resistance in HER2-enriched patients [36,62].

The presence of *PIK3CA* gene mutations has been observed in approximately 9% of TNBC, including cases that recur as metastatic tumors after initial HR-positive BC [63]. In such cases, the *PIK3CA* mutation has been found to persist [63]. TNBC can be classified into six subtypes according to gene expression, as proposed by Lehman [32]. Among these subtypes, the luminal androgen receptor (LAR) and mesenchymal stem-like (MSL) subtypes exhibit a greater frequency of *PIK3CA* mutations [32]. The role of *PIK3CA* mutations and alterations in the PI3K/AKT pathway is significant in BC biology. However, their significance is more comprehensively understood in HR-positive/HER2-negative BC in comparison to TNBC and HER2-enriched BC, which necessitate additional research endeavors [8].

In the clinical setting of TNBC and HER2-enriched subtypes, it is important to highlight their aggressive features. These types of BC demonstrate wild-type status in relation to *PIK3CA* mutations. The presence of other complex pathways that have substantial roles in the development of BC across different molecular subtypes may explain this phenomenon. In order to advance future research, it is crucial to conduct further investigation into the PIK3/AKT/PTEN and mTOR pathways, as they are closely associated with *PIK3CA*. Moreover, it is imperative to conduct protein expression level analysis to obtain a comprehensive understanding of the involvement of these pathways in TNBC and HER2-enriched subtypes.

Our study also has limitations. We lacked certain clinical variables such as staging, metastasis status, and tumor-infiltrating lymphocytes, mainly because we relied on archival data. This limitation prevented us from analyzing how PIK3CA mutations correlate with these factors and from following patients for long-term prognoses. Prior research shows that AR-positive TNBC cell lines respond significantly to PI3K inhibitors combined with AR antagonists, suggesting a biomarker-driven approach for selecting TNBC patients [32]. Regrettably, our study focuses on the fundamental molecular subtypes of TNBC, not the additional six types proposed by Lehman [32]. Future work should incorporate these refined subtyping methods to better discern the role of *PIK3CA* mutations in TNBC and explore novel therapy combinations.

## Conclusion

Our research underscores a high frequency of the *PIK3CA* H1047L mutation in Indonesian BC, with luminal B HER2-negative and luminal A subtypes showing the highest rates. Furthermore, the location of the mutated *PIK3CA* samples were associated with age groups, with the majority of *PIK3CA* exon 9 mutations identified in individuals aged ≤ 50 years and *PIK3CA* exon 20 mutations in older patients. These findings prompt further exploration of the role of *PIK3CA* mutations as biomarkers for personalized therapy, particularly in hormone receptor-positive disease.

## References

[1] Giaquinto AN, Sung H, Miller KD, Kramer JL, Newman LA, Minihan A, et al. Breast Cancer Statistics, 2022. CA Cancer J Clin. 2022;72(6):524–41. doi: 10.3322/caac.21754 36190501

[2] Wilkinson L, Gathani T. Understanding breast cancer as a global health concern. Br J Radiol. 2022;95(1130):20211033. doi: 10.1259/bjr.20211033 34905391 PMC8822551

[3] Bray F, Laversanne M, Sung H, Ferlay J, Siegel RL, Soerjomataram I, et al. Global cancer statistics 2022: GLOBOCAN estimates of incidence and mortality worldwide for 36 cancers in 185 countries. CA Cancer J Clin. 2024;74(3):229–63. doi: 10.3322/caac.21834 38572751

[4] Smolarz B, Nowak AZ, Romanowicz H. Breast Cancer-Epidemiology, Classification, Pathogenesis and Treatment (Review of Literature). Cancers (Basel). 2022;14(10):2569. doi: 10.3390/cancers14102569 35626173 PMC9139759

[5] Feng Y, Spezia M, Huang S, Yuan C, Zeng Z, Zhang L, et al. Breast cancer development and progression: Risk factors, cancer stem cells, signaling pathways, genomics, and molecular pathogenesis. Genes Dis. 2018;5(2):77–106. doi: 10.1016/j.gendis.2018.05.001 30258937 PMC6147049

[6] Alowiri NH, Hanafy SM, Haleem RA, Abdellatif A. PIK3CA and PTEN Genes Expressions in Breast Cancer. Asian Pac J Cancer Prev. 2019;20(9):2841–6. doi: 10.31557/APJCP.2019.20.9.2841 31554385 PMC6976819

[7] Cizkova M, Susini A, Vacher S, Cizeron-Clairac G, Andrieu C, Driouch K, et al. PIK3CA mutation impact on survival in breast cancer patients and in ERα, PR and ERBB2-based subgroups. Breast Cancer Res. 2012;14(1):R28. doi: 10.1186/bcr3113 22330809 PMC3496146

[8] Hu H, Zhu J, Zhong Y, Geng R, Ji Y, Guan Q, et al. PIK3CA mutation confers resistance to chemotherapy in triple-negative breast cancer by inhibiting apoptosis and activating the PI3K/AKT/mTOR signaling pathway. Ann Transl Med. 2021;9(5):410. doi: 10.21037/atm-21-698 33842631 PMC8033310

[9] Miricescu D, Totan A, Stanescu-Spinu I-I, Badoiu SC, Stefani C, Greabu M. PI3K/AKT/mTOR Signaling Pathway in Breast Cancer: From Molecular Landscape to Clinical Aspects. Int J Mol Sci. 2020;22(1):173. doi: 10.3390/ijms22010173 33375317 PMC7796017

[10] Martínez-Sáez O, Chic N, Pascual T, Adamo B, Vidal M, González-Farré B, et al. Frequency and spectrum of PIK3CA somatic mutations in breast cancer. Breast Cancer Res. 2020;22(1):45. doi: 10.1186/s13058-020-01284-9 32404150 PMC7222307

[11] Vatte C, Al Amri AM, Cyrus C, Chathoth S, Alsayyah A, Ahmad A, et al. Helical and kinase domain mutations of PIK3CA, and their association with hormone receptor expression in breast cancer. Oncol Lett. 2019;18(3):2427–33. doi: 10.3892/ol.2019.10565 31404155 PMC6676675

[12] Keraite I, Alvarez-Garcia V, Garcia-Murillas I, Beaney M, Turner NC, Bartos C, et al. PIK3CA mutation enrichment and quantitation from blood and tissue. Sci Rep. 2020;10(1):17082. doi: 10.1038/s41598-020-74086-w 33051521 PMC7555501

[13] Schagerholm C, Robertson S, Toosi H, Sifakis EG, Hartman J. PIK3CA mutations in endocrine-resistant breast cancer. Sci Rep. 2024;14(1):12542. doi: 10.1038/s41598-024-62664-1 38822093 PMC11143214

[14] Verret B, Cortes J, Bachelot T, Andre F, Arnedos M. Efficacy of PI3K inhibitors in advanced breast cancer. Ann Oncol. 2019;30 Suppl 10:x12–20. doi: 10.1093/annonc/mdz381 31859349 PMC6923787

[15] Wang M, Li J, Huang J, Luo M. The Predictive Role of PIK3CA Mutation Status on PI3K Inhibitors in HR+ Breast Cancer Therapy: A Systematic Review and Meta-Analysis. Biomed Res Int. 2020;2020:1598037. doi: 10.1155/2020/1598037 32461963 PMC7238354

[16] Kim JW, Lim AR, You JY, Lee JH, Song SE, Lee NK, et al. PIK3CA Mutation is Associated with Poor Response to HER2-Targeted Therapy in Breast Cancer Patients. Cancer Res Treat. 2023;55(2):531–41. doi: 10.4143/crt.2022.221 36097803 PMC10101795

[17] Wu H, Wang W, Du J, Li H, Wang H, Huang L, et al. The distinct clinicopathological and prognostic implications of PIK3CA mutations in breast cancer patients from Central China. Cancer Manag Res. 2019;11:1473–92. doi: 10.2147/CMAR.S195351 30863158 PMC6388997

[18] Al-Shamsi HO, Abu-Gheida I, Abdulsamad AS, AlAwadhi A, Alrawi S, Musallam KM, et al. Molecular Spectra and Frequency Patterns of Somatic Mutations in Arab Women with Breast Cancer. Oncologist. 2021;26(11):e2086–9. doi: 10.1002/onco.13916 34327780 PMC8571745

[19] Hamadeh LN, Farhat L, Hilal L, Assi H, Nasr F, Chahine G, et al. Frequency and mutational spectrum of PIK3CA gene mutations in breast cancer patients: Largest and first report from Lebanon. Gene. 2023;871:147433. doi: 10.1016/j.gene.2023.147433 37068694

[20] Ben Rekaya M, Sassi F, Saied E, Bel Haj Kacem L, Mansouri N, Zarrouk S, et al. PIK3CA mutations in breast cancer: A Tunisian series. PLoS One. 2023;18(5):e0285413. doi: 10.1371/journal.pone.0285413 37195967 PMC10191322

[21] Lian J, Xu E-W, Xi Y-F, Wang H-W, Bu P, Wang J-F, et al. Clinical-Pathologic Analysis of Breast Cancer With PIK3CA Mutations in Chinese Women. Technol Cancer Res Treat. 2020;19:1533033820950832. doi: 10.1177/1533033820950832 33047659 PMC7557680

[22] Deng L, Zhu X, Sun Y, Wang J, Zhong X, Li J, et al. Prevalence and Prognostic Role of PIK3CA/AKT1 Mutations in Chinese Breast Cancer Patients. Cancer Res Treat. 2019;51(1):128–40. doi: 10.4143/crt.2017.598 29540052 PMC6333988

[23] Lee M-H, Cho J-H, Kwon S-Y, Jung S-J, Lee J-H. Clinicopathological Characteristics of PIK3CA Mutation and Amplification in Korean Patients with Breast Cancers. Int J Med Sci. 2020;17(8):1131–5. doi: 10.7150/ijms.44319 32410843 PMC7211160

[24] Elwy F, Helwa R, El Leithy AA, Shehab El din Z, Assem MM, Hassan NHA. PIK3CA mutations in HER2-positive Breast Cancer Patients; Frequency and Clinicopathological Perspective in Egyptian Patients. Asian Pac J Cancer Prev. 2017;18(1):57–64. doi: 10.22034/APJCP.2017.18.1.57 28240010 PMC5563120

[25] De Mattos-Arruda L. PIK3CA mutation inhibition in hormone receptor-positive breast cancer: time has come. ESMO Open. 2020;5(4):e000890. doi: 10.1136/esmoopen-2020-000890 32817061 PMC7437706

[26] Kalinsky K, Jacks LM, Heguy A, Patil S, Drobnjak M, Bhanot UK, et al. PIK3CA mutation associates with improved outcome in breast cancer. Clin Cancer Res. 2009;15(16):5049–59. doi: 10.1158/1078-0432.CCR-09-0632 19671852

[27] Zardavas D, Fumagalli D, Loi S. Phosphatidylinositol 3-kinase/AKT/mammalian target of rapamycin pathway inhibition: a breakthrough in the management of luminal (ER+/HER2-) breast cancers? Curr Opin Oncol. 2012;24(6):623–34. doi: 10.1097/CCO.0b013e328358a2b5 22960556

[28] Mosele F, Stefanovska B, Lusque A, Tran Dien A, Garberis I, Droin N, et al. Outcome and molecular landscape of patients with PIK3CA-mutated metastatic breast cancer. Ann Oncol. 2020;31(3):377–86. doi: 10.1016/j.annonc.2019.11.006 32067679

[29] Lv W, Du C, Zhang Y, Wu F, Jin Y, Chen X, et al. Clinicopathological characteristics and prognostic analysis of PIK3CA mutation in breast cancer patients in Northwest China. Pathol Res Pract. 2022;238:154063. doi: 10.1016/j.prp.2022.154063 35994807

[30] Ishida N, Baba M, Hatanaka Y, Hagio K, Okada H, Hatanaka KC, et al. PIK3CA mutation, reduced AKT serine 473 phosphorylation, and increased ERα serine 167 phosphorylation are positive prognostic indicators in postmenopausal estrogen receptor-positive early breast cancer. Oncotarget. 2018;9(25):17711–24. doi: 10.18632/oncotarget.24845 29707142 PMC5915150

[31] Tharin Z, Richard C, Derangère V, Ilie A, Arnould L, Ghiringhelli F, et al. PIK3CA and PIK3R1 tumor mutational landscape in a pan-cancer patient cohort and its association with pathway activation and treatment efficacy. Sci Rep. 2023;13(1):4467. doi: 10.1038/s41598-023-31593-w 36934165 PMC10024711

[32] Lehmann BD, Bauer JA, Schafer JM, Pendleton CS, Tang L, Johnson KC, et al. PIK3CA mutations in androgen receptor-positive triple negative breast cancer confer sensitivity to the combination of PI3K and androgen receptor inhibitors. Breast Cancer Res. 2014;16(4):406. doi: 10.1186/s13058-014-0406-x 25103565 PMC4187324

[33] McGee SR, Tibiche C, Trifiro M, Wang E. Network Analysis Reveals A Signaling Regulatory Loop in the PIK3CA-mutated Breast Cancer Predicting Survival Outcome. Genomics Proteomics Bioinformatics. 2017;15(2):121–9. doi: 10.1016/j.gpb.2017.02.002 28392480 PMC5414713

[34] Ramirez-Ardila D, Timmermans AM, Helmijr JA, Martens JWM, Berns EMJJ, Jansen MPHM. Increased MAPK1/3 Phosphorylation in Luminal Breast Cancer Related with PIK3CA Hotspot Mutations and Prognosis. Transl Oncol. 2017;10(5):854–66. doi: 10.1016/j.tranon.2017.08.002 28886403 PMC5591392

[35] Garay JP, Smith R, Devlin K, Hollern DP, Liby T, Liu M, et al. Sensitivity to targeted therapy differs between HER2-amplified breast cancer cells harboring kinase and helical domain mutations in PIK3CA. Breast Cancer Res. 2021;23(1):81. doi: 10.1186/s13058-021-01457-0 34344439 PMC8336338

[36] Cizkova M, Dujaric M-E, Lehmann-Che J, Scott V, Tembo O, Asselain B, et al. Outcome impact of PIK3CA mutations in HER2-positive breast cancer patients treated with trastuzumab. Br J Cancer. 2013;108(9):1807–9. doi: 10.1038/bjc.2013.164 23612454 PMC3658522

[37] Pang B, Cheng S, Sun S-P, An C, Liu Z-Y, Feng X, et al. Prognostic role of PIK3CA mutations and their association with hormone receptor expression in breast cancer: a meta-analysis. Sci Rep. 2014;4:6255. doi: 10.1038/srep06255 25176561 PMC4150110

[38] Cizkova M, Cizeron-Clairac G, Vacher S, Susini A, Andrieu C, Lidereau R, et al. Gene expression profiling reveals new aspects of PIK3CA mutation in ERalpha-positive breast cancer: major implication of the Wnt signaling pathway. PLoS One. 2010;5(12):e15647. doi: 10.1371/journal.pone.0015647 21209903 PMC3012715

[39] Guo S, Loibl S, von Minckwitz G, Darb-Esfahani S, Lederer B, Denkert C. PIK3CA H1047R Mutation Associated with a Lower Pathological Complete Response Rate in Triple-Negative Breast Cancer Patients Treated with Anthracycline-Taxane-Based Neoadjuvant Chemotherapy. Cancer Res Treat. 2020;52(3):689–96. doi: 10.4143/crt.2019.497 32019278 PMC7373870

[40] Heriyanto DS, Yoshuantari N, Akbariani G, Lau V, Hanini H, Hidayati Z, et al. High Probability of Lynch Syndrome Among Colorectal Cancer Patients Is Associated With Higher Occurrence of KRAS and PIK3CA Mutations. World J Oncol. 2024;15(4):612–24. doi: 10.14740/wjon1843 38993255 PMC11236368

[41] Khoury K, Tan AR, Elliott A, Xiu J, Gatalica Z, Heeke AL, et al. Prevalence of Phosphatidylinositol-3-Kinase (PI3K) Pathway Alterations and Co-alteration of Other Molecular Markers in Breast Cancer. Front Oncol. 2020;10:1475. doi: 10.3389/fonc.2020.01475 32983983 PMC7489343

[42] Rahmawati Y, Setyawati Y, Widodo I, Ghozali A, Purnomosari D. Molecular Subtypes of Indonesian Breast Carcinomas - Lack of Association with Patient Age and Tumor Size. Asian Pac J Cancer Prev. 2018;19(1):161–6. doi: 10.22034/APJCP.2018.19.1.161 29373908 PMC5844611

[43] Tau S, Miller TW. Alpelisib Efficacy without Cherry-PI3King Mutations. Clin Cancer Res. 2023;29(6):989–90. doi: 10.1158/1078-0432.CCR-22-3411 36626159 PMC10023365

[44] Fujimoto Y, Morita TY, Ohashi A, Haeno H, Hakozaki Y, Fujii M, et al. Combination treatment with a PI3K/Akt/mTOR pathway inhibitor overcomes resistance to anti-HER2 therapy in PIK3CA-mutant HER2-positive breast cancer cells. Sci Rep. 2020;10(1):21762. doi: 10.1038/s41598-020-78646-y 33303839 PMC7729878

[45] Hayama S, Nakamura R, Ishige T, Sangai T, Sakakibara M, Fujimoto H, et al. The impact fpostmenopausal luminal breast cancer patients. BMC Cancer. 2023;23: 1–10. doi: 10.1186/S12885-023-10853-Y/TABLES/6 37106324 PMC10134571

[46] Saikia KK, Panigrahi MK, Mehta A, Kumar D. Clinico-pathological Features of PIK3CA Mutation in HER2-Positive Breast Cancer of Indian Population. Indian J Surg Oncol. 2018;9(3):381–6. doi: 10.1007/s13193-018-0749-3 30288002 PMC6154351

[47] Abba MC, Zhong Y, Lee J, Kil H, Lu Y, Takata Y, et al. DMBA induced mouse mammary tumors display high incidence of activating Pik3caH1047 and loss of function Pten mutations. Oncotarget. 2016;7(39):64289–99. doi: 10.18632/oncotarget.11733 27588403 PMC5325442

[48] Xiao W, Zhang G, Chen B, Chen X, Wen L, Lai J, et al. Mutational Landscape of PI3K-AKT-mTOR Pathway in Breast Cancer: Implications for Targeted Therapeutics. J Cancer. 2021;12(14):4408–17. doi: 10.7150/jca.52993 34093841 PMC8176410

[49] Zhao Y, Tang W, Sun L, Xiong D, Sheng M, Luo Y. The effects of PIK3CA and TP53 on prognosis in patients with Triple-negative breast cancer (TNBC): a single institution’s long-term follow-up (6 years). European Journal of Gynaecological Oncology. 2021;42(6):1320. doi: 10.31083/j.ejgo4206191

[50] Loi S, Haibe-Kains B, Majjaj S, Lallemand F, Durbecq V, Larsimont D, et al. PIK3CA mutations associated with gene signature of low mTORC1 signaling and better outcomes in estrogen receptor-positive breast cancer. Proc Natl Acad Sci U S A. 2010;107(22):10208–13. doi: 10.1073/pnas.0907011107 20479250 PMC2890442

[51] Bertucci A, Bertucci F, Gonçalves A. Phosphoinositide 3-Kinase (PI3K) Inhibitors and Breast Cancer: An Overview of Current Achievements. Cancers (Basel). 2023;15(5):1416. doi: 10.3390/cancers15051416 36900211 PMC10001361

[52] Yip PY. Phosphatidylinositol 3-kinase-AKT-mammalian target of rapamycin (PI3K-Akt-mTOR) signaling pathway in non-small cell lung cancer. Transl Lung Cancer Res. 2015;4(2):165–76. doi: 10.3978/j.issn.2218-6751.2015.01.04 25870799 PMC4384220

[53] Spangle JM, Von T, Pavlick DC, Khotimsky A, Zhao JJ, Roberts TM. PIK3CA C-terminal frameshift mutations are novel oncogenic events that sensitize tumors to PI3K-α inhibition. Proc Natl Acad Sci U S A. 2020;117(39):24427–33. doi: 10.1073/pnas.2000060117 32929011 PMC7533832

[54] Peixoto A, Cirnes L, Carvalho AL, Andrade MJ, Brito MJ, Borralho P, et al. Evaluation of PIK3CA mutations in advanced ER+/HER2-breast cancer in Portugal - U-PIK Project. Front Mol Biosci. 2023;10:1082915. doi: 10.3389/fmolb.2023.1082915 36825198 PMC9941536

[55] Cancer Genome Atlas Network. Comprehensive molecular portraits of human breast tumours. Nature. 2012;490(7418):61–70. doi: 10.1038/nature11412 23000897 PMC3465532

[56] Berns K, Horlings HM, Hennessy BT, Madiredjo M, Hijmans EM, Beelen K, et al. A functional genetic approach identifies the PI3K pathway as a major determinant of trastuzumab resistance in breast cancer. Cancer Cell. 2007;12(4):395–402. doi: 10.1016/j.ccr.2007.08.030 17936563

[57] Keegan NM, Furney SJ, Walshe JM, Gullo G, Kennedy MJ, Smith D, et al. Phase Ib Trial of Copanlisib, A Phosphoinositide-3 Kinase (PI3K) Inhibitor, with Trastuzumab in Advanced Pre-Treated HER2-Positive Breast Cancer “PantHER”. Cancers (Basel). 2021;13(6):1225. doi: 10.3390/cancers13061225 33799597 PMC7999809

[58] Nakai M, Yamada T, Sekiya K, Sato A, Hankyo M, Kuriyama S, et al. Use of Liquid Biopsy to Detect PIK3CA Mutation in Metastatic Breast Cancer. J Nippon Med Sch. 2022;89(1):66–71. doi: 10.1272/jnms.JNMS.2022_89-107 33692304

[59] Wu H-J, Chu P-Y. Current and Developing Liquid Biopsy Techniques for Breast Cancer. Cancers (Basel). 2022;14(9):2052. doi: 10.3390/cancers14092052 35565189 PMC9105073

[60] Galvano A, Castellana L, Gristina V, La Mantia M, Insalaco L, Barraco N, et al. The diagnostic accuracy of PIK3CA mutations by circulating tumor DNA in breast cancer: an individual patient data meta-analysis. Ther Adv Med Oncol. 2022;14:17588359221110162. doi: 10.1177/17588359221110162 36188485 PMC9516428

[61] Dumbrava EE, Call SG, Huang HJ, Stuckett AL, Madwani K, Adat A, et al. PIK3CA mutations in plasma circulating tumor DNA predict survival and treatment outcomes in patients with advanced cancers. ESMO Open. 2021;6(5):100230. doi: 10.1016/j.esmoop.2021.100230 34479035 PMC8414046

[62] Rugo HS, Raskina K, Schrock AB, Madison RW, Graf RP, Sokol ES, et al. Biology and Targetability of the Extended Spectrum of PIK3CA Mutations Detected in Breast Carcinoma. Clin Cancer Res. 2023;29(6):1056–67. doi: 10.1158/1078-0432.CCR-22-2115 36321996 PMC10011882

[63] Pascual J, Turner NC. Targeting the PI3-kinase pathway in triple-negative breast cancer. Ann Oncol. 2019;30(7):1051–60. doi: 10.1093/annonc/mdz133 31050709

